# Effect of ethanolic extract of propolis as an alternative to antibiotics as a growth promoter on broiler performance, serum biochemistry, and immune responses

**DOI:** 10.14202/vetworld.2017.249-254

**Published:** 2017-02-24

**Authors:** Abbasali Gheisari, Shekofa Shahrvand, Nasir Landy

**Affiliations:** 1 Animal Science Research Department, Isfahan Agricultural and Natural Resources Research and Education Center, AREEO, Isfahan, Iran; 2 Department of Animal Science, Isfahan (Khorasgan) Branch, Islamic Azad University, Isfahan, Iran; 3 Young Researchers and Elite Club, Isfahan (Khorasgan) Branch, Islamic Azad University, Isfahan, Iran

**Keywords:** antibiotic, broilers, growth performance, immune responses, propolis, serum biochemistry

## Abstract

**Aim:**

An *in vivo* experiment was conducted to investigate the effect of different levels of ethanolic extract of propolis, on growth performance, carcass traits, serum biochemistry, and humoral immune responses of chickens, as compared with the antibiotic flavophospholipol.

**Materials and Methods:**

312 1-day-old as-hatched broiler chicks (Ross 308) were randomly allotted to 6 treatments with 4 replicate pens per treatment. The 6 dietary treatments fed for 42 days consisted of a corn-soybean meal basal diet (control); control plus 4.5 mg/kg flavophospholipol, and control plus 50, 100, 200, and 300 mg/kg ethanol extracts of propolis, respectively.

**Results:**

Neither propolis nor antibiotic affected the performance criteria; however, dietary treatments tended to enhance to enhance body weight and daily feed intake of broiler chickens compared with control group (p>0.05). None of the dietary treatments significantly altered feed: Gain though; broilers fed diet supplemented with 200 mg/kg propolis had better feed: gain values compared with other groups in starter, and grower phases as well as the whole experimental period (p>0.05). Carcass yield and internal organ relative weights were not affected by treatments on day 42, except for abdominal fat pad weight that decreased in broilers supplemented with antibiotic. None of the treatments significantly affected humoral immune function. Dietary treatments failed to induce any significant effect on serum biochemistry (p>0.05); though broilers receiving 100 mg/kg propolis had greater high-density lipoprotein-cholesterol and lower triglyceride concentrations compared with other groups.

**Conclusion:**

In conclusion, the results indicated that addition of ethanolic extract of propolis to routine dietary components of broilers, such as corn and soybean, seems not to have a positive influence on performance criteria.

## Introduction

Antibiotic growth promoters (AGPs) have been used for around 60 years in agricultural animal production to enhance growth performance and protect health of animals [1-7]. AGPs have been supposed to enhance growth performance of poultry because it changes the intestinal tract, and hence, improving absorptive capacity [8]. However, there are concerns that wider subtherapeutic use of AGP in animal feed can lead to the development of antibiotic resistance in human pathogens, which is considered as a potential risk for humans if it is transferred from animal to human microbiota [9,10]. Thus, poultry producers are looking for strategies to allow them to reduce or eliminate the use of AGP in poultry production. As a consequence, there is growing demand for alternatives to the subtherapeutic use of antibiotics which are able to sustain or improve broiler performance and the safety of poultry products.

Propolis is a sticky gummy resinous material that worker honeybees (*Apis mellifera*) collect from young shoots and buds of certain trees and shrubs and mix with wax and salivary enzymes [11]. Propolis possesses flavonoids, aromatic acids, caffeic acids, terpenes, and phenolic compounds with proven various biological activities such as antibacterial [12-14], antiviral [15], antifungal [16], anti-inflammatory [17], analgesic and tissue regenerative [18], antioxidant [19,20], and cytostatic and hepatoprotective activities [19].

Khojasteh and Shivazad [21] reported the beneficial effects of ethanolic extract of propolis on broilers performance indices but later in another study Kleczek *et al*. [22] observed no significant effect of propolis on broiler performance or carcass traits. Eyng *et al*. [23] reported a negative effect of feeding ethanolic extract of propolis to broilers on growth performance during the starter period and found no significant differences at 42 days of age. Caffeic acid and quercetin isolated from propolis did not seem to affect antibody production in rats [12]. On the other hand, Freitas *et al*. [24] showed that supplementation of propolis to laying hens enhanced the production of immunoglobulin (Ig) G specific to sheep red blood cell (SRBC) and natural antibodies and could be used to increase antigen-specific antibody responses to vaccines.

The objective of this study was to evaluate the effects of ethanolic extract of propolis on the growth performance, carcass traits, serum biochemistry, and humoral immune responses in broiler chickens.

## Materials and Methods

### Ethical approval

The Animal Care Committee of University of Isfahan reviewed and approved all procedures performed in the trial.

### Propolis preparation

Propolis was collected from hive of honey bee via plastic nets. A 30% propolis solution was prepared by mixing 700 ml of 96% ethyl alcohol and 300 g of propolis. The solution was kept in a container, in the absence of bright light, at room temperature and it was shaken twice a day. After 2 weeks, the extract was filtered, and final concentration was evaporated by vacuum evaporator at 35°C.

### Determination of total phenolic contents

The total polyphenols in the ethanolic extract of propolis were determined according to the method described by Pierpoint [25].

### Animals and dietary treatments

312 1-day-old as-hatched Ross 308 broilers were weighed at the time of arrival and randomly assigned to 6 treatments, each with 4 replicate pens of 13 chicks. The following treatments were applied: A corn-soybean meal basal diet containing no additives (Table-1) as the negative control (C), the diet C + 4.5 mg flavophospholipol/kg as a positive control, or the diet C + 50, 100, 200 or 300 mg ethanolic extract of propolis/kg of diet.

**Table-1:** The ingredient and calculated composition of basal starter, and grower diets (as-fed basis).

Item	Starter (1-21 days)	Grower (22-42 days)
Ingredient (as fed), g/kg		
Corn	560	580
Soybean meal 44% CP	389	350
Soybean oil	10	34
Mono-calcium (15% Ca), phosphate (23% P)	13	9.3
CaCO_3_	17.3	17
NaCl	3.5	3.3
Trace mineral premi×^1^	2.7	2.7
Vitamin premi×^2^	2.7	2.7
DL-methionine	1.8	1
Calculated composition, g/kg		
Metabolizable energy, kcal/kg	2825	3030
CP	204	189
Calcium	9.8	9.0
Available phosphorus	4.4	3.5
Methionine+cysteine	8.7	7.4
Lysine	11.9	10.9

1 The mineral premix provided the following per kilogram of diet: 120 mg of Zn from ZnSO_4_, 120 mg of Mn from MnSO_4_, 80 mg of Fe from FeSO_4_5H_2_O, 10 mg of Cu from CuSO_4_, 2.5 mg of I from CaIO_4_, 1 mg of Co from CoSO_4_, and 0.2 mg of Se from Na_2_SeO_3_.

2 The vitamin premix provided the following per kilogram of diet: 13,200 IU of vitamin A, 4,000 ICU of vitamin D, 66 IU of vitamin E, 39.6 µg of vitamin B_12_, 13.2 mg of riboflavin, 110 mg of niacin, 22 mg of D-pantothenate, 0.4 mg of vitamin K; 2.2 mg of folic acid, 4.0 mg of thiamin; 7.9 mg of pyridoxine, 0.253 mg biotin, and 100 mg of ethoxyquin. CP=Crude protein

The basal diet was formulated to meet or exceed the NRC (1994) nutrient specifications for broilers [26]. The feeding program included a starter diet from 1 to 21 days (2825 kcal ME/kg, 20.4% crude protein [CP]), followed by grower diet from 22 to 42 days (3,030 kcal ME/kg, 18.9% CP). The trial was conducted in pens (120 cm × 120 cm × 80 cm) for 6 weeks and water and feed were provided *ad libitum* intake throughout the entire trial period. The photoschedule consisted of a period of 23-h light and 1 h of darkness for the duration of the experiment. Broilers were kept in a temperature-controlled house at 32°C from day 1 to 7, 29°C for day 8 to 14, 26°C for day 15 to 21, and 22° for day 22 to the end of the trial.

### Performance and carcass components

Body weights (BW) and mortality of broilers were recorded at 21 and 42 days of age. Daily weight gain (DWG) and daily feed intake (DFI) were measured at the end of weeks 3 and 6, and feed conversion ratio defined as DFI/DWG (g: g) was calculated accordingly.

At 42 days of age, 2 male birds from each replicate were randomly chosen, placed in transportation coops, weighed, and then killed by cervical dislocation. Carcass yields were determined as the carcass weight (free from the head, feet, abdominal fat pad, and viscera) in relation to live weight. Abdominal fat, heart, liver, pancreas, and cecum weights were determined and expressed as a percentage of BW.

### Immunity

At 9 days of age, broiler chicks were administered subcutaneously on the dorsal region of the neck with 0.2 ml of the Newcastle disease (NDV) and avian influenza (AI; subtype H9) inactivated vaccine and live vaccine strain Lasota of the NDV at 21 days of age (orally). Antibody titers against NDV, avian influenza virus (AIV), and SRBC, and heterophil to lymphocyte (H: L) ratio were measured as immune responses. At 25 days of age, 2 cockerels within each replicate were inoculated intravenously with 1 ml of 1% SRBC. Six days after injection, chicks were bled and antibody titers were determined. Total SRBC antibody was expressed as the log_2_ of the reciprocal of the last dilution which agglutination was observed [27]. At 28 days of age, blood was obtained from 2 cockerels and plasma was tested for detecting antibody to NDV and AIV antigens, via the hemagglutination inhibition methods (HI), HI antibody titer of the serum was converted to log_2_.

At 42 days of age, 8 birds per treatment were used for determining H: L ratio. Blood samples were taken from brachial veins using syringes containing heparin as anticoagulant. Blood smears were prepared using May-Grunwald-Giemsa stain [28]. The number of H and L were counted to a total of 60 cells, and the H: L ratio was calculated [29].

### Serum biochemistry

At 42 days of age, after 12 h of fasting, approximately 2 ml of blood per bird was collected via brachial vein with commercial vacuum tubes into tubes without lithium heparin and incubated at 37°C for 2 h, centrifuged at 2000 ×*g* at 8°C for 10 min (SIGMA 4-15 Lab Centrifuge, Germany) and serum was separated for biochemical analysis. Two replicate serum samples per pen were analyzed for triglyceride, total cholesterol, high-density lipoprotein (HDL) and low-density lipoprotein (LDL), and cholesterol using the kit package (Pars Azmoon Co; Tehran, Iran).

### Statistical analysis

The data were subjected to analysis of variance procedures appropriate for a completely randomized design using the general linear model procedures of SAS [30]. Means were compared using Tukey test. Statements of statistical significance are based on p<0.05.

## Results

### Total phenolic content

The ethanolic extract used in this trial contained 173.6 mg/g of total polyphenols.

### Performance and carcass traits

Data on performance indices are summarized in Table-2. Broiler BW did not differ between the experimental treatments at days 21 (starter period), though it tended to enhance in broilers fed diets containing antibiotic or different levels of ethanolic extract of propolis. Similarly, during the grower phase (22-42 days), BW of broilers was not statistically affected by the treatments, though it tended to increase in broilers fed diets containing antibiotic or different levels of ethanolic extract of propolis. There were no significant differences in DFI between treatments, during starter period. Treatments did not induce any significant impact on DFI in growth period as well as the whole experiment (1-42 days) although broilers fed diets supplemented with antibiotic or different levels of ethanolic extract of propolis had a marginally higher DFI in comparison with the control group. During starter and grower phases as well as for the whole trial, broilers fed diet supplemented with 200 mg/kg ethanolic extract of propolis had better feed: Gain values compared with other groups, whereas the results were not statistically significant. No significant (p>0.05) differences due to treatment effects were observed on mortality.

**Table-2:** Effect of experimental diets on performance indices of broilers at different ages.

Item	Experimental treatment						SEM

	Control	Antibiotic	50 ppm propolis	100 ppm propolis	200 ppm propolis	300 ppm propolis	
BW, g							
Day 21	617.5	632.1	613.5	646.5	634.6	618.6	17.2
Day 42	2164	2252	2248	2285	2189	2223	63.8
DFI, g/day							
Day 1-21	45.2	45.7	45.7	46.4	45.1	45.7	0.7
Day 22-42	147.3	152.1	154.0	154.0	144.3	153.8	5.85
Day 1-42	95.7	99.1	100.1	100.2	99.6	98.5	3.9
Feed: gain, g: g							
Day 1-21	1.66	1.63	1.63	1.62	1.60	1.66	0.1
Day 22-42	2.00	1.97	2.00	2.00	1.94	2.01	0.05
Day 1-42	1.89	1.87	1.88	1.91	1.84	1.91	0.04

SEM=Standard error of mean, BW=Body weight, DFI=Daily feed intake

Table-3 shows relative weight means of organs as a percentage of live weight. In the current trial, carcass yield and internal organs relative weights were not significantly affected by the dietary treatments except for relative weight of abdominal fat pad that decreased in broilers supplemented with antibiotic.

**Table-3:** Effect of experimental treatments on carcass yield and internal relative organ weight of broilers at 42 days of age.

Relative organ weight (%)	Experimental treatment						SEM

	Control	Antibiotic	50 ppm propolis	100 ppm propolis	200 ppm propolis	300 ppm propolis	
Carcass,	70.0	70.1	69.19	71.0	69.1	71.1	1.15
Abdominal fat,	1.53^ab^	1.34^b^	1.83^a^	1.66^ab^	1.56^ab^	1.64^ab^	0.23
Liver,	2.19	2.33	2.34	2.39	2.29	2.14	0.20
Heart,	0.56	0.63	0.55	0.60	0.58	0.58	0.04
Pancreas,	0.23	0.24	0.25	0.24	0.24	0.24	0.04
Cecum,	0.58	0.53	0.62	0.64	0.62	0.63	0.10

^a,b^Values in the same row not sharing a common superscript differ (p<0.05). SEM=Standard error of mean

### Immune responses

The effect of experimental treatments on humoral immune responses is presented in Table-4. This should be rephrased to “there was no effect of dietary treatment on immune related parameters assessed including antibody titers against NDV, AI, SRBC, and H: L ratio treatments.”

**Table-4:** Effect of experimental treatments on antibody titers against Newcastle and Influenza viruses at 28 days of age and SRBC at 31 days of age, and H/L and albumin to globulin ratios at 42 days of age.

Item	Experimental treatment						SEM

	Control	Antibiotic	50 ppm propolis	100 ppm propolis	200 ppm propolis	300 ppm propolis	
New castle (log_2_)	7.25	7.10	6.75	6.36	6.72	6.90	0.5
Influenza (log_2_)	5.25	4.50	5.35	4.72	5.36	4.60	0.75
SRBC (log_2_)	7.75	6.80	7.15	6.54	7.27	7.10	0.87
H/L	0.44	0.51	0.48	0.46	0.49	0.46	0.04

SEM=Standard error of mean, H/L=Heterophil to lymphocyte ratio, SRBC=Sheep red blood cells

### Serum biochemistry

Table-5 summarizes the impact of treatments on serum constituents at day 42 of age. None of the serum biochemical parameters tested were significantly affected by the experimental treatments (p>0.05); though broilers receiving 100 mg/kg ethanolic extract of propolis had higher HDL-cholesterol and lower triglyceride concentrations compared with other groups.

**Table-5:** Effect of dietary treatments on serum biochemical parameters of broilers at 42 days of age.

Item	Experimental treatment						SEM

	Control	Antibiotic	50 ppm propolis	100 ppm propolis	200 ppm propolis	300 ppm propolis	
Triglyceride, mg/100 mL	73	56	61	57	65	73	9.50
Total cholesterol, mg/100 mL	98	106	106	111	97	111	9.03
LDL-cholesterol, mg/100 mL	26	29	29	32	28	31	4.60
HDL-cholesterol, mg/100 mL	79	81	84	97	77	88	11.20

SEM=Standard error of mean, LDL=Low-density lipoprotein, HDL=High-density lipoprotein

## Discussion

In this study, neither antibiotic nor propolis affected the performance of broilers. Similar to our results, Kleczek *et al*. [22] reported that use of Flavomycin or propolis failed to have any effect on performance of broilers, which is in contrast with the findings of Attia *et al*. [31] who reported that use of propolis continuously or intermittently enhanced BW and improved feed: Gain during the entire period of the study in comparison with the control. Roodsari *et al*. [32] reported that dietary supplementation of propolis increased final BW and improved feed: Gain compared with those fed basal diet. In this study, there was no significant effect of supplementation with flavophospholipol. This is in contrast to the response reported by other authors [1,2,33,34]. Coates *et al*. [35] reported that supplementation of broiler chickens with antibiotics in a germ-free environment could not improve growth as compared to those raised in conventional environment, leading the researchers to conclude that antibiotics decreased growth of pathogenic bacteria responsible for growth depression. Thus, it seems that in our study propolis or antibiotic could not induce any positive effects on growth performance due to the hygienic status of the trial.

In this study, there was no significant effect of experimental treatment on carcass traits except for abdominal fat pad, which decreased in broilers supplemented with antibiotic. Our findings on carcass characteristics are also in accord to those of Torki *et al*. [36] who did not report any significant influence of ethanol extract of propolis on the relative weights of the breast, legs, liver, heart, abdominal fat pad and gall bladder, at slaughter age in broilers. Denli *et al*. [37] also reported that there were no significant differences in relative weight of carcass, abdominal fat, liver, gizzard, proventriculus, and small intestine of quails fed diets supplemented with different levels of propolis. Seven *et al*. [38] reported that administration of ethanol extract of propolis could improve carcass yield of broilers raised under heat stress condition. In this trial, the lack of significant influence of the propolis on carcass traits could be attributed to the highly digestible basal diet and/or the ideal conditions of the experiment.

There was not effect of treatment on immune related parameters in this trial. The antibody levels are some indices for humoral immune responses; so, the results indicated that the supplementation of ethanolic extract of propolis was not effective for improving humoral immunity in birds. In accordance with our results, Eyng *et al*. [39] reported that the addition of 1-4% of propolis extraction residue containing polyphenols and flavonoids to broiler diets was not capable of promoting immune responses; however, several trials have indicated that propolis is able to improve Ig production [40-42] and can be used as adjuvant in vaccines to improve immunogenicity. It seems that in the current trial ethanolic extract of propolis could not increase antibody titers because of the levels used were not enough to improve immune responses of broiler chicks.

Fuliang *et al*. [43] reported that supplementation of ethanol and water extracts of propolis decreased total cholesterol, triglyceride, LDL-cholesterol, very LDL cholesterol, and increased HDL-cholesterol in serum of fasting rats. Denli *et al*. [37] reported that broilers received Turkish propolis in the diet tended to have higher serum HDL and lower serum LDL. The enhancement of serum HDL cholesterol by addition of 100 mg/kg ethanolic extract of propolis observed in the current trial might be due to the reduction of synthetic enzyme activities. However, further research is needed to clarify the mechanism of hypolipidemic actions of propolis.

## Conclusion

In this experiment, there was no significant effect of dietary supplementation with ethanolic extract of propolis on performance of broiler chickens. Supplementation with ethanolic extract of propolis resulted in favorable effects on blood chemistry although the results were not statistically significant. The results of this trial suggest that further research is justified.

## Authors’ Contributions

AG and SS have designed the plan of work. SS carried out the laboratory work and analyzed the results. AG and NL drafted and revised the manuscript. All authors have read and approved the final manuscript.

## References

[1] Landy N, Ghalamkari G. H, Toghyani M (2011a). Performance, carcass characteristics, and immunity in broiler chickens fed dietary neem (*Azadirachta indica*) as alternative for an antibiotic growth promoter. Livest. Sci.

[2] Landy N, Ghalamkari G. H, Toghyani M, Moattar F (2011b). The effects of *Echinacea purpurea* L. (purple coneflower) as an antibiotic growth promoter substitution on performance, carcass characteristics and humoral immune response in broiler chickens. J. Med. Plants Res.

[3] Landy N, Ghalamkari G. H, Toghyani M (2012). Evaluation of St. John’s Wort (*Hypericum perforatum* L.) as an antibiotic growth promoter substitution on performance, carcass characteristics, some of the immune responses, and serum biochemical parameters of broiler chicks. J. Med. Plants Res.

[4] Yazdi F. F, Ghalamkari G. H, Toghiani M, Modaresi M, Landy N (2014a). Anise seed (*Pimpinella anisum* L.) as an alternative to antibiotic growth promoters on performance, carcass traits and immune responses in broiler chicks. Asian Pac. J. Trop. Dis.

[5] Yazdi F. F, Ghalamkari G. H, Toghiani M, Modaresi M, Landy N (2014b). Efficiency of *Tribulus terrestris* L. as an antibiotic growth promoter substitute on performance and immune responses in broiler chicks. Asian Pac. J. Trop. Dis.

[6] Nanekarani S, Goodarzi M, Heidari M, Landy N (2012). Efficiency of ethanolic extract of peppermint (*Mentha piperita*) as an antibiotic growth promoter substitution on performance, and carcass characteristics in broiler chickens. Asian Pac. J. Trop. Biomed.

[7] Goodarzi M, Nanekarani S. H, Landy N (2014). Effect of dietary supplementation with onion (*Allium cepa* L.) on performance, carcass traits and intestinal microflora composition in broiler chickens. Asian Pac. J. Trop. Dis.

[8] Visek W. J (1978). The mode of growth promotion by antibiotics. J. Anim. Sci.

[9] Nasir Z, Grashorn M. A (2006). Effects of Intermittent Application of Different *Echinacea purpurea* Juices on Broiler Performance and Some Blood Indices. PhD Dissertation.

[10] Toghyani M, Mosavi S. K, Modaresi M, Landy N (2015). Evaluation of kefir as a potential probiotic on growth performance, serum biochemistry and immune responses in broiler chicks. Anim. Nutr.

[11] Greenaway W, Scaysbrook T, Whatley F. R (1990). The composition and plant origins of propolis. Bee World.

[12] Sforcin J. M, Orsi R. O, Bankova V (2005). Effect of propolis, some isolated compounds and its source plant on antibody production. J. Ethnopharmacol.

[13] Kujumgiev A, Tsvetkova I, Serkedjieva Y, Bankova V, Christov R, Popov S (1999). Antibacterial, antifungal and antiviral activity of propolis of different geographic origin. J. Ethnopharmacol.

[14] Silici S, Kutluca S (2005). Chemical composition and antibacterial activity of propolis collected by three different races of honeybees in the same region. J. Ethnopharmacol.

[15] Vynograd N, Vynograd I, Sosnowski Z (2000). A comparative multicentre study of the efficacy of propolis, acyclovir and placebo in the treatment of genital herpes (HSV). Phytomedicine.

[16] Ota C, Unterkircher C, Fantinato V, Shimizu M. T (2001). Antifungal activity of propolis on different species of candida. Mycoses.

[17] Orsatti C. L, Missima F, Pagliarone A. C, Sforcin J. M (2010). Th1/Th2 cytokines’expression and production by propolis-treated mice. J. Ethnopharmacol.

[18] De Castro S. L (2001). Propolis: Biological and pharmacological activities. Therapeutic uses of this bee product. Annu. Rev. Biomed. Sci.

[19] Banskota A. H, Tezuka Y, Adnyana I. K, Midorikawa K, Matsushige K, Message D, Huertas A. A, Kadota S (2000). Cytotoxic, hepatoprotective and free radical scavenging effects of propolis from Brazil, Peru, the Netherlands and China. J. Ethnopharmacol.

[20] Orhan H, Marol S, Hepsen I. F, Sahin G (1999). Effects of some probable antioxidants on selenite-induced cataract formation and oxidative stress-related parameters in rats. Toxicology.

[21] Khojasteh S, Shivazad M (2006). The effect of diet propolis supplementation on ross broiler chicks performance. Int. J. Poult. Sci.

[22] Kleczek K, Wilkiewicz-Wawro E, Wawro K, Makowski W, Murawska D, Wawro M (2014). The effect of dietary propolis supplementation on the growth performance of broiler chickens. Pol. J. Nat. Sci.

[23] Eyng C, Murakami A. E, Duarte C. R, Santos T. C (2014). Effect of dietary supplementation with an ethanolic extract of propolis on broiler intestinal morphology and digestive enzyme activity. J. Anim. Physiol. Anim. Nutr.

[24] Freitas J. A, Vanat N, Pinheiro J. W, Balarin M. R. S, Sforcin J. M, Venancio E. J (2011). The effects of propolis on antibody production by laying hens. Poult. Sci.

[25] Pierpoint W. S (2004). The extraction of enzymes from plant tissues rich in phenolic compounds. Methods Mol. Biol.

[26] NRC (1994). Nutrient Requirements of Poultry.

[27] Wegmann T. G, Smithies O (1966). A simple hemagglutination system requiring small amounts of red blood cells and antibodies. Transfusion.

[28] Lucas A. M, Jamroz C (1961). Atlas of Avian Hematology.

[29] Gross W. B, Siegel P. S (1983). Evaluation of heterophil to lymphocyte ratio as a measure of stress in chickens. Avian Dis.

[30] SAS Institute (1996). SAS/STAT^®^ User’s Guide: Statistics, Version 6.12.

[31] Attia Y. A, Al-Hamid A. E. A, Ibrahim M. S, Al-Harthi M. A, Bovera F, Elnaggar A. S. H (2014). Productive performance, biochemical and hematological traits of broiler chickens supplemented with propolis, bee pollen, and mannan oligosaccharides continuously or intermittently. Livest. Sci.

[32] Roodsari M. H, Mehdizadeh M, Kasmani F. B, Lotfelahian H, Mosavi F, Abolghasemi A. H (2004). Effects of oil-extracted propolis on the performance of broiler chicks. Agric. Sci. Technol.

[33] Kavyani A, Shahne A. Z, PorReza J, Haji-abadi S. M. A, Landy N (2012). Evaluation of dried powder of mushroom (*Agaricus bisporus*) as an antibiotic growth promoter substitution on performance, carcass traits and humoral immune responses in broiler chickens. J. Med. Plants Res.

[34] Landy N, Kavyani A (2014). Effect of using multi-strain probiotic on performance, Immune responses, and cecal microflora composition in broiler chickens reared under heat stress condition. Iran. J. Appl. Anim. Sci.

[35] Coates M. E, Fuller R, Harrison G. F, Lev M, Suffolk S. F (1963). A comparison of the growth of chicks in the gustafsson germ-free apparatus and in a convectional environment, with and without dietary supplements of penicillin. Br. J. Nutr.

[36] Torki M, Soltani J, Mohammadi H (2015). Effects of adding ethanol extract of propolis and cumin essential oil to diet on the performance, blood parameters, immune response and carcass traits of broiler chicks. Iran. J. Appl. Anim. Sci.

[37] Denli M, Cankaya S, Silici S, Okan F, Uluocak A. N (2005). Effect of dietary addition of Turkish propolis on the growth performance, carcass characteristics and serum variables of quail (*Coturnix coturnix japonica*). Asian Aust. J. Anim. Sci.

[38] Seven P. T, Seven I, Yılmaz M, Simsşek U. G (2008). The effects of Turkish propolis on growth and carcass characteristics in broilers under heat stress. Anim. Feed Sci. Technol.

[39] Eyng C, Murakami A. E, Santos T. C, Silveira T. G, Pedroso R. B, Lourenço D. A (2015). Immune responses in broiler chicks fed propolis extraction residue-supplemented diets. Asian Aust. J. Anim. Sci.

[40] Ziaran H. R, Rahmani H. R, Pourreza J (2005). Effect of dietary oil extract of propolis on immune response and broiler performance. Pak. J. Biol. Sci.

[41] (2010). Çetin E, Silici S, Çetin, N. and Güçlü B K. (. Poult. Sci.

[42] Daneshmand A, Sadeghi G. H, Karimi A, Vaziry A, Ibrahim S. A (2015). Evaluating complementary effects of ethanol extract of propolis with the probiotic on growth performance, immune response and serum metabolites in male broiler chickens. Livest. Sci.

[43] Fuliang H. U, Hepburn H. R, Xuan H, Chen M, Daya S, Radloff S. E (2005). Effects of propolis on blood glucose, blood lipid and free radicals in rats with diabetes mellitus. Pharmacol. Res.

